# Voices of Women in Mathematical Sciences Higher Education: the PhD Student Experience

**DOI:** 10.1007/s44007-026-00256-3

**Published:** 2026-11-04

**Authors:** Kaitlyn N. Ries, Alexander J. Martin, Laura E. Wadkin

**Affiliations:** 1 School of Mathematics, Statistics and Physics, Newcastle University, Newcastle upon Tyne, NE1 7RU UK; 2 Faculty of Medical Sciences, Newcastle University, Newcastle upon Tyne, NE1 7RU UK

**Keywords:** Women, Mathematics, Mathematical sciences, Doctoral students, Barriers to progression, Leaky pipeline, Lived experiences, PhD student

## Abstract

Women remain under-represented in the mathematical sciences across UK Higher Education Institutions (HEIs). In this study we focus on the doctoral student experience in relation to gender to identify factors impacting the retention of women in academia post-PhD. Using qualitative methods, we explore the lived experiences of female PhD students within a mathematical sciences department at a UK HEI. Through in-depth one-to-one interviews, we explore participant perceptions of how gender has impacted their PhD experiences and shaped their academic journeys, including their relationships with supervisors, exposure to role models, identity as a mathematician, and career plans beyond the PhD. We identify six recurring themes: a sense of not belonging, feeling treated differently by supervisors, a lack of exposure to female role models, experiences of microaggressions and sexism, a complex picture of professional self-identity, and the perception of gender as a barrier to an academic career. We also explore participants’ recommendations for improving research culture. This work deepens our understanding of the lived experiences of female PhD students in male-dominated fields and will further inform the development of targeted interventions to enhance the academic environment for women in the mathematical sciences.

**Supplementary Information:**

The online version contains supplementary material available at https://doi.org/10.1007/s44007-026-00256-3.

## Introduction

Women remain under-represented in the mathematical sciences and other STEM (Science, Technology, Engineering and Mathematics) disciplines within higher education across Europe and globally [1–4]. This gender imbalance intensifies throughout successive stages of academic career progression and is often referred to as the “leaky pipeline” [5], with male-dominance most pronounced in permanent academic research and teaching positions. In the mathematical sciences in the UK, this is starkly reflected in The London Mathematical Society National Benchmarking Study [6] which reports that in 2021/22, while 38% of undergraduates and 46% of master’s students were female, just 33% of doctoral (PhD) students, 26% of researchers and lecturers, and 14% of professors were female. These figures show minimal improvement since the original survey in 2018 [7].

While gender imbalances persist throughout academic STEM disciplines, doctoral study represents a particularly important stage within the academic pipeline [4, 8]. The PhD functions as a transition between education and an academic career, during which students develop as independent researchers, establish professional identities, build academic networks, and make decisions about their future careers [9–11]. Consequently, experiences during doctoral study influence whether individuals remain both within the field of mathematical sciences and within academia itself [8, 11, 12].

A comprehensive and holistic understanding of the lived experiences of women and other gender minorities in mathematical sciences higher education is necessary to address the persistent gender imbalance in the field. Qualitative research techniques are particularly suited to this task, offering rich insights into the lived experiences of individuals, allowing the exploration of their thoughts, feelings, beliefs and behaviours [13, 14]. Existing qualitative studies have examined gender-related barriers at various stages of the STEM academic pipeline, including early engagement in science [15], the undergraduate experience [16, 17], PhD participation [18], career progression [19, 20] and academic leadership [21].

More recent work in the UK and Ireland continues to highlight the importance of understanding lived experiences in shaping educational and career trajectories. For example, the ASPIRES longitudinal research programme at University College London has examined how young adults aged 20–23 in STEM fields make educational and career decisions, and how these choices are shaped by gender and intersecting factors such as social class and ethnicity [22]. In Ireland, a study with female STEM students used in-depth interviews to explore undergraduate and postgraduate experiences [23]. Other recent reports have presented the impact of caring responsibilities on STEM researchers [24], the influence of gender on publication activity [25], and the career expectations of recent undergraduates [26].

While these studies have provided valuable insights into the experiences and aspirations of women in STEM, important gaps remain. Much of the existing research focuses on broad STEM populations, often combining participants from multiple disciplines, which limits our understanding of experiences that may be specific to the mathematical sciences and its disciplinary culture. There has also been more focus on the undergraduate student experience, with comparatively little research with PhD students, specifically within UK higher education contexts.

In this study, we focus specifically on the lived experiences of female PhD students in the mathematical sciences within a UK higher education institution (HEI). Using qualitative methods, we explore how gender shapes their doctoral experiences, identify recurring themes within their narratives, and examine their recommendations for improving research culture for women in a traditionally male-dominated field. By focusing on an underexplored stage of the mathematical sciences pipeline, this work provides a mathematics-specific account of women’s doctoral experiences and highlights participant-led recommendations for improving research culture and supporting women’s progression within the discipline. The following section reviews the existing literature that motivates the study before we outline the methodology, results, limitations, and conclusions.

## Lived Experiences of Women in Mathematics

The persistent underrepresentation of women in mathematics and the wider STEM disciplines has motivated research seeking to understand the factors that influence participation and retention. While quantitative studies are valuable in identifying patterns of representation and progression across educational and career stages [6, 7], qualitative approaches provide deeper insights into the lived experiences behind these trends [13, 14, 27–30]. A growing body of qualitative research has examined the experiences of women across the STEM pipeline globally, which has revealed several recurring themes within women’s narratives.

### Belonging

A sense of belonging, the feeling of being accepted, valued, and included within a group or setting, is of fundamental importance for psychological wellbeing [31]. “Not belonging” is prevalent in the accounts of women in STEM throughout the academic career stages and is recognised as an important factor influencing both progression and retention. In the US, research has shown that women who remain in STEM careers report stronger feelings of belonging than those who leave [32] and that sense of belonging is a predictor of college students’ intent to pursue mathematics-related careers [33].

Challenges to belonging appear throughout the educational pipeline. Qualitative studies of undergraduate mathematical sciences students have documented feelings of exclusion, marginalisation, and visibility as being ‘‘different’’ from their peers [34, 35]. Undergraduate participants in one UK-based study described feeling simultaneously ‘‘invisible’’ while remaining highly visible (“special”) as women in a male-dominated field [16]. Similar findings have been reported among women studying mathematics-intensive STEM subjects, including engineering and computer science [23, 36–39]. These experiences may be influenced by disciplinary gender balance, with women more likely to report a sense of belonging in STEM subjects with greater female representation [32].

Studies of doctoral STEM students in the US have shown that reduced belonging is associated with poorer wellbeing and lower persistence, with women often reporting lower levels of belonging than their male peers [40–42]. Similar findings have been reported in other global contexts, with peer social support linked to promoting researcher identity development in Australia [9] and belonging associated with wellbeing and engagement in social science and humanities postgraduate students in the UK [43]. Together, these findings suggest that belonging remains a considerable factor in doctoral education. However, there is limited research exploring how women undertaking mathematics doctoral studies experience belonging in specifically UK higher education contexts.

### Relationships, Mentoring, and Support

Relationships with peers, supervisors, mentors, and role models are widely recognised as important influences on students’ experiences and progression within STEM disciplines [9, 44–47].

Previous work has demonstrated the importance of female role models, suggesting that they improve the academic performance, persistence, and professional identity of women in STEM [48–51]. Female role models may also strengthen belonging by challenging stereotypes regarding who can succeed within traditionally male-dominated disciplines [32, 52]. Exposure to women in positions of academic success therefore provides an important mechanism through which women can envision themselves within STEM careers. Similarly, female mentors have been shown to positively influence both mentees [19, 53] and the mentors themselves [46].

At the doctoral level, supervisory relationships represent a particularly important source of academic and professional support. Previous research has demonstrated that positive supervisory relationships play an essential role in cultivating a positive doctoral experience [10] and in increasing retention [8, 11, 12], well-being [45, 54, 55] and student satisfaction [12, 54].

Whilst the value of female role models in male-dominated environments is well established, studies examining supervisor gender have reported inconsistent findings. A study of US chemistry PhD graduates found that female students supervised by women were more likely to become faculty themselves [56]. In contrast, other work has shown that gender-matching of doctoral students and supervisor does not necessarily improve the experience for women [57], and instead suggests that the personal and professional qualities of the supervisor are more impactful than supervisor gender [58]. Nevertheless, increasing access to female supervisors remains challenging in disciplines where women are underrepresented, and may risk placing disproportionate demands on existing female academics [59–61].

In addition to supervisory and mentoring relationships, peer networks have been shown to play an important role in STEM doctoral students' experiences, fostering belonging, providing emotional and academic support, and contributing to the development of researcher identity [9]. A study of science and engineering doctoral students in Taiwan found that peer mentors provided instrumental, psychosocial and emotional support, while also helping students adapt to the culture of their research groups and academic communities [62].

Collectively, the literature demonstrates that relationships and support networks play a central role in shaping women’s experiences within STEM. However, much of the existing evidence comes from broad STEM populations or non-UK contexts. Consequently, relatively little is known about how women undertaking doctoral study specifically within the mathematical sciences experience and draw upon these different forms of support.

### Careers, Job Security, and Family Commitments

Decisions regarding future academic careers are shaped not only by students' experiences within their departments but also by perceptions of the wider academic career structure. In the US, family formation was suggested as the largest barrier between postgraduate studies and faculty positions for women in science [63]. Similarly, in the UK and Ireland, gendered expectations around parenthood, limited paid parental leave, and unclear maternity leave policies have been identified as disadvantaging to female academics across disciplines [64, 65]. A recent survey also highlighted the difficulty academics with caring responsibilities face in undertaking career development activities involving travel, such as conferences [24].

These concerns are particularly prominent in the narratives of PhD and post-doctoral researchers across countries and disciplines who already face instabilities due to temporary contracts and job insecurity [58, 64, 66, 67]. Concerns around casualisation remain prominent within UK academia, with recent industrial action drawing attention to the widespread use of fixed-term contracts for researchers [68]. Such precarity has been identified as a barrier to an academic career in both UK higher education and globally [67, 69] and disproportionately impacts those with caring responsibilities, often women [64, 70, 71].

Research suggests that supportive institutional environments can mitigate some of these challenges. Flexible working arrangements and support for work-life balance have been associated with increased job satisfaction and a sense of workplace belonging across genders, career stage and STEM disciplines [72]. Both male and female PhD students in a US study reported experiencing less stress and greater career commitment when faculty in their departments validate the importance of personal relationships and family commitments and actively support students in finding a healthy work-life balance [57]. Despite ongoing efforts to improve practice, developing effective approaches to supporting researchers with job precarity and caring responsibilities remains an important challenge for UK HEIs [73, 74].

### Identity, Confidence, Stereotypes, and Sexism

Professional and academic identity can broadly be understood as how individuals perceive themselves and their place within their professional and academic communities, including whether they view themselves as legitimate members of their discipline [75]. Previous research across various countries has highlighted the importance of identity development for women in STEM [76–78] and in academia more generally [79].

For doctoral students, this involves a transition from student to independent researcher and the gradual development of identities such as ‘‘scientist’’, ‘‘researcher’’, or ‘‘mathematician’’ [80]. This process is shaped not only by academic achievement but also by social relationships, institutional cultures, and perceptions of who belongs within a discipline [43, 75–77]. Identity development is further influenced by the interaction of gender with other social identities and lived experiences, as illustrated by research with minority ethnic women in UK STEM higher education [81].

Within male-dominated STEM fields, professional identity development can be complicated by persistent gender stereotypes. Qualitative studies have shown that women frequently encounter cultural narratives that associate mathematical ability, scientific brilliance, and technical expertise with masculinity [16, 77, 82–84]. These stereotypes may influence how women perceive their own abilities and whether they feel recognised by others as legitimate members of the discipline. Studies of women in the mathematical sciences have highlighted tensions between participants' identities and the stereotypical images of mathematicians, often requiring them to challenge assumptions linking mathematical competence with masculinity [16, 83, 85, 86].

A recurring theme within the literature is confidence and imposter syndrome [87]. Characterised by persistent doubts about one’s abilities despite evidence of success, imposter syndrome is pervasive in the narratives of women across academic STEM [23, 88–92]. It appears to be most common amongst women in fields that are male-dominated [93] and that are “perceived to require brilliance”, including mathematics [84]. At doctoral level, imposter syndrome has been associated with intensified feelings of inadequacy, challenges to academic identity development and a reduced sense of belonging [89, 93, 94].

Women may also experience pressure to navigate competing expectations surrounding gender and professional identity, often conceptualised through stereotype threat, where awareness of negative stereotypes can influence behaviour, performance, and self-perception [95]. Female mathematicians have reported disavowing feminine characteristics which are stereotypically negatively associated with success, including expressing a desire to have children [96]. In several studies in the US and Canada, female STEM PhD students report “dressing in a less feminine manner” to “blend in” to their male-dominated environment [97], and female doctoral physics students said “looking girly” would undermine their identity as a physicist [98]. Such experiences are perhaps unsurprising given evidence that feminine appearance is negatively associated with perceptions of scientific identity [99].

Experiences of sexism and microaggressions have been highlighted by women throughout the academic STEM pipeline [19, 100–104]. Microaggressions are subtle, often everyday behaviours, comments, or interactions that communicate negative or stereotypical assumptions about an individual's social group, regardless of whether harm is intended [105]. These experiences have been shown to contribute to a decreased sense of belonging [102, 106], hinder leadership progression [101] and decrease job satisfaction [107, 108]. Much recent research has focussed on the forms and impacts of gender-based microaggressions [101, 103, 104, 109]. Together, these studies suggest that the cumulative effect of seemingly minor incidents can influence women's sense of belonging, professional identity, and perceptions of their future within academia.

In response to these challenges, previous work has explored interventions aimed at improving research culture and reducing the effects of sexism and microaggressions. Allyship from both male and female colleagues has been identified as an important mechanism for mitigating the impact of gendered microaggressions [101], while interventions designed to raise awareness of implicit bias have shown promise in supporting longer-term cultural change [110]. More broadly, frameworks for addressing sexism in academia emphasise the importance of challenging structural inequalities, promoting awareness and reflection, and ensuring diverse voices are represented in institutional decision-making [111]. Many HEIs now prioritise equality, diversity and inclusion (EDI) initiatives [112–115], and engagement with the global Athena Swan Charter, which provides a framework for promoting gender equality [114]. While there is evidence that the Athena Swan charter has resulted in improvements in research culture [116, 117], challenges remain in translating institutional commitments into meaningful improvements in the lived experiences of women academics [118].

### Summary of the Literature and Rationale for the Present Study

Collectively, the literature discussed above demonstrates that women's experiences in STEM are shaped by a complex interaction of experiences and perceptions. Across educational and career stages, women consistently report challenges relating to belonging, access to role models and mentors, confidence and professional identity, and the navigation of gendered academic cultures.

However, important limitations remain within the existing literature. Much of the research has focused on undergraduate students or established researchers, despite doctoral study representing a critical transition point between formal education and an academic career. Many studies focus on other disciplines or combine participants from a broad range of STEM disciplines, making it difficult to understand experiences that may be specific to the mathematical sciences. Third, a substantial proportion of the literature originates from the US or other international contexts, with comparatively little work exploring the lived experiences of women undertaking doctoral study within UK mathematical sciences departments [119].

To address these gaps, this study uses qualitative methods to explore the lived experiences of female doctoral students within a UK mathematical sciences department. By focusing on an underexplored stage of the academic pipeline, it provides insight into how gender shapes doctoral experiences and offers evidence to inform efforts to improve research culture and support women's progression within the mathematical sciences.

## Materials and Methods

### Researcher Positionality

The research team consisted of three academics with different disciplinary backgrounds and career stages. At the time of data collection and analysis, KNR was a master’s student in Mathematics and Statistics, AJM was a medical doctor with experience in medical education, and LEW was a research fellow in applied mathematics. KNR and LEW are women with experience of studying and working within mathematical sciences environments, while AJM brought expertise in widening participation research from outside the discipline and in medical educational studies. These backgrounds provided valuable insight into the context under study but also inform the lenses through which participants' accounts were interpreted [120, 121].

KNR and LEW's lived experiences provided familiarity with many of the issues discussed by participants, including experiences of underrepresentation, belonging, confidence, role models, and gendered interactions within academic environments. This disciplinary and personal proximity may have increased sensitivity to these themes during data collection and analysis but also introduced the possibility that participants' experiences could be interpreted through the researchers’ own assumptions and experiences. The involvement of a researcher from outside the mathematical sciences provided an additional perspective that helped challenge disciplinary assumptions during theme development.

### Institutional Context

The study was conducted within a mathematical sciences department at a large research-intensive Russell Group university in the north of England with approximately 27,000 students and 6000 staff members. The university itself has a broadly balanced student population, with women comprising approximately half of all students and doctoral researchers. The mathematical sciences department comprises undergraduate, postgraduate taught, doctoral, and academic staff populations with researchers and students spanning pure mathematics, applied mathematics, statistics, data science, and physics fields. All doctoral students within these areas were eligible to participate. In common with the wider mathematical sciences sector, women are underrepresented within the department. At the time of the study, the department contained approximately 100 academic staff (23% female), 80 postgraduate research (PhD) students (30% female), 10 postgraduate taught (master’s) students (60% female) and 850 undergraduates (33% female) and has remained similar over recent years. Most funded doctoral positions in the department are awarded through a competitive selection process involving application, interview, and assessment of prior academic achievement (as described below).

### UK Higher Education Context

For international readers, it is important to note that students in the UK typically specialise earlier than in many other higher education systems. Undergraduate degree programmes normally focus on a single discipline and are typically completed over three years. Doctoral (PhD) programmes are research-focused degrees, generally lasting three to four years. Entry to doctoral study in mathematical sciences fields typically requires a strong undergraduate degree (at least an upper second-class honours bachelor’s degree) and most entrants have completed a master’s degree, although this is not always a formal requirement.

### Participants

Current or recently graduated PhD students who identified as female or as a member of a gender minority group were eligible to participate. Consistent with the departmental structure, doctoral students from pure mathematics, applied mathematics, statistics, data science, and physics were all eligible for inclusion. Participants were recruited through departmental email advertisements distributed during April and May 2022. The study used self-selection sampling—participation was voluntary, and all eligible respondents who expressed an interest in taking part were included in the study.

### Survey and Interviews

Participants completed a survey one week prior to their interview. The survey provided background information and encouraged participants to begin reflecting on their PhD experiences before the interview. One-hour online interviews were subsequently conducted in June and July 2022 to explore the participants’ lived experiences and identify potential interventions that could improve the experiences of women in the mathematical sciences.

The interview schedule was developed following a review of the literature on women’s experiences in mathematics and STEM, with input from a university representative on EDI. Topics included issues identified as important in previous research, such as belonging, role models, confidence, gender stereotypes, sexism, and career aspirations. These topics were used as prompts to support discussion while allowing participants substantial freedom to introduce experiences and perspectives that they considered important.

Interviews followed a semi-structured format, using a combination of open-ended questions and prompts that encouraged participants to reflect on and interpret their experiences. The interviews followed a broadly chronological structure, beginning with school experiences before moving through undergraduate and doctoral study. Participants were encouraged to guide the discussion and to introduce issues they felt were relevant to their experiences. The interviews sought to provide participants with opportunities to describe their experiences in their own terms and raise issues of personal importance. While some of the themes ultimately identified aligned with those reported in earlier studies, the analysis provided an opportunity to explore how these issues were experienced by women undertaking doctoral study within the mathematical sciences.

A copy of both the pre-interview survey and the interview guide is provided in the Supplementary Materials as Online Resource 1 and Online Resource 2 respectively.

### Thematic Analysis

Reflexive thematic analysis was chosen as the analytical approach for this study due to its flexibility in identifying and interpreting patterns within qualitative data [120, 121]. Although the interview schedule was informed by previous literature, coding was not organised around the interview topics themselves. Rather, themes were developed inductively through iterative engagement with participants' accounts, allowing recurring patterns to be identified across the dataset.

Interviews were automatically transcribed, manually checked for accuracy, and uploaded to NVivo 14 for thematic analysis. KNR and LEW independently coded the transcripts, identifying both explicit and implicit meanings within participants' accounts. Codes were iteratively reviewed, refined, and grouped into broader themes through discussion and repeated engagement with the data to produce a coherent thematic structure [120, 121]. Throughout the analysis, emerging themes were discussed collaboratively within the research team, alternative interpretations were considered, and repeated reference was made to the original transcripts to ensure that findings remained grounded in participants' accounts rather than researchers' prior expectations (see Researcher positionality).

### Ethical Approval, Consent, and Data Management

This study was approved by the Newcastle University Faculty of Science, Agriculture and Engineering Ethics Committee (Reference: 19,881, 10/03/2022).

Participants were briefed on the intention of the study and the data management plan through a Participant Information Sheet. All participants supplied written informed consent. Participants were free to withdraw from the study at any time without giving any reason for withdrawal. All participants who were consented for inclusion received a £25 book voucher as a thanks for their participation.

The participants consented to video recording of the online interview to allow transcription by the authors, after which the recordings were permanently deleted. Information that could lead to identification of participants has been anonymised.

## Results

Eight participants were recruited. Five were current PhD students (with one in their first year, two in their second, and two in their third) and three had graduated within the last year. All participants self-identified their gender as female, with no participants identifying as transgender, non-binary or any other gender minority. The participants’ research areas spanned pure mathematics, applied mathematics, physics, and statistics. Six participants had completed their undergraduate studies at UK HEIs and two completed their undergraduate studies internationally.

In this section we detail the recurring themes in the participants’ PhD experiences, identified through a thematic analysis of their one-to-one interviews, and present their suggestions for improving research culture.

### Recurring Themes

All but one participant felt that overall, their gender had a direct negative impact on their PhD experiences. Six recurring themes were identified across the participants’ narratives which contributed to this perception:

- A sense of not belonging.
- Feeling “treated differently” by supervisors.
- A lack of exposure to female role models.
- Experiences of microaggressions and sexism.
- A complex picture of professional self-identity.
- Gender as a barrier to an academic career.

In the following sections we will explore these themes through direct quotes from the participants.

#### A Sense of Not Belonging

We explored PhD student participants’ sense of belonging to their PhD peer group, their HEI mathematical sciences department, and the wider mathematical community. This was explored both through direct questioning and indirectly, through discussions about their sense of acceptance and inclusion.

For most participants, self-examination of their belonging began during their mathematics A-level studies (ages 16–18). One participant’s teachers were “all really surprised that I picked maths and physics for A-level because I was a girl”. For some, a gender imbalance was apparent at this stage: “I was surprised when I got there that I was the only one or two [women]. It was kind of like the other girls had been told something I hadn’t. I did feel like a little bit of an outsider.” Others, however, reported that their A-level classes were “a pretty even split”, resulting in them “[not] noticing anything to do with gender”.

Throughout their undergraduate studies, most participants perceived an even gender balance at first: “It did seem pretty equal overall, particularly in the earlier years, I can’t remember noticing any particular gender split.” However, many noted differences between mathematics subspecialties, reporting that “in some modules I was the only girl” and “the more statistics modules [seemed] to have a better [gender] balance”, while “in physics there were definitely more guys”. One participant said they felt the reason for this was “the perception that [physics and applied maths] are harder than statistics and pure”. This was exacerbated in later years: “Particularly in my [final year], I pretty much only took applied maths modules. [There] were only two girls in the class […] that felt weird.” This gender-imbalance contributed to a feeling of “standing out”, which could be intimidating: “I definitely felt more scared of the modules that were more male dominated.” It even influenced some participants’ module choices: “It was one of the reasons I didn’t do any applied stuff, and I really should have done […] I regret it now.”

While some participants began to question their belonging at an early stage, this was most apparent during PhD study. Participants felt they noticed unexpected differences in gender balance between different research groups: “I had no idea that some areas of [astrophysics] were more male dominated than others” and “I still notice that [research group] is basically entirely men.” One participant highlighted that their own research group felt “more diverse”, speaking positively of colleagues highlighting their preferred choice of pronouns in email signatures and talks, whilst “no one from [another research group] has pronouns […] it seems a much more heteronormative, straight male dominated group”. For many, this had a direct impact on their sense of belonging: “Gender wise, I don’t feel like I belong in that research group.” One participant said, “[I was] standing out for the wrong reasons, it didn’t seem to matter how well I understood the subject […] I was an outsider”, whilst another described feeling a greater sense of belonging in another research group with a more even gender balance: “Gender and diversity wise, I feel more like I belong there […] my work belongs in [research group 1], but gender wise I belong in [research group 2].” Some reported these feelings leading to missed educational and networking opportunities: “I don't even want to go to [seminars], because it just seems like a less approachable group.”

While many participants spoke positively of their relationships with peers in their PhD cohort (“among my peers, I feel like I belong”), some felt a lack of inclusion, saying that they “keep themselves to themselves”, “don’t fit in”, and “feel like an outsider”. One participant said, “On the social side […] there’s a big group of guys who will go out to the pub, and I just don’t fancy being the only woman.” Additionally, participants described experiences of gender-related microaggressions and sexism from their PhD peers (discussed in further detail below).

Many participants reported developing a stronger sense of belonging over the course of their PhD. One participant reflected “I did have difficulty fitting in [at first], but I think I do feel I belong now.” While awareness of gender imbalance and gendered challenges remained, some participants described becoming increasingly confident and assertive in claiming their place within the department: “I do feel I belong there, whether anybody else would agree with that, I don’t care. I feel I belong there.”

#### Feeling “Treated Differently” by Supervisors

All participants in this study had either two or three supervisors, all of whom were male. Most participants spoke positively of their supervisory relationships in general: “I have a very good relationship with both of them […] they are challenging when I make mistakes and they are supportive when I do good things.” Participants also reported feeling supported in both research and pastoral capacities. One participant highlighted the emotional support available from her supervisor, explaining that “if I am having a bad week, or something is going on in my life, I can confide in [my supervisor]”, while another described receiving “constant advice on what sort of career [and] what sort of directions I need to take if I want to either continue on the path of research or if I want to leave academia”.

Despite this, many perceived that in some respects they had still been treated differently by their male supervisors due to their gender. One participant felt that she did not have the same relationship with her supervisor as male students, explaining that “I don’t feel like we're as close as male-male student-supervisors.” She noted that her supervisor had “a [male] PhD student who they were really close to” and that “when they go away and do work trips together that sounds very different to when we go away and do trips”. She speculated that this difference could reflect her supervisor’s concerns about how the relationship may be perceived by others: “[Observers] might think ‘they look very close’, and somebody would assume something [inappropriate], just because we're male and female.” Another participant echoed these concerns, saying she felt that male supervisors “feel more comfortable to be pally with guys”.

Even when describing positive relationships with male supervisors, some participants reflected on whether gender might shape those interactions. One participant commented that “It’s obviously good that he is nice to me, but I do wonder if it’s because I’m a woman, and he’s more… gentle.” Another similarly reflected that although peers described her supervisor as someone who “can be a bit mean”, “he’s always been really nice to me, and I don’t know if that’s just a personal thing or it’s because I’m a woman”.

When asked explicitly if they felt that having a female academic as part of their supervisory team would be beneficial, most participants agreed, suggesting that their experience navigating a male-dominated field “would be closer to mine than a man’s would”. However, some participants emphasised that broader female representation within their research environment was more important than the gender of their supervisor alone. As one participant explained, “It would be fine to have someone just in the field or in the wider department.”

#### A Lack of Exposure to Female Role Models

Encouragingly, participants identified female mathematics teachers at school as “wonderful” and “encouraging” role models who made it seem “normal for a woman to be good at maths”. For one participant, “I never questioned whether I could do [mathematics] because they were doing it.” This is in stark contrast to their undergraduate experiences, where it was apparent from “early on” that they would have “only one or two female lecturers” throughout their whole degree programme.

At PhD level, participants described a continuing lack of female role models and mentors. Beyond providing academic guidance, role models were viewed as important sources of support for understanding and contextualising gendered experiences. As one participant explained, *“*If you're struggling with something […] it just seems like there are no women to talk to you about it.*”* Another reflected, *“*I would love to talk to a woman who's been in this position and be like, ‘do you think it's a gender thing?*’”*

Participants also emphasised the importance of seeing women occupying positions they themselves might one day aspire to. One participant explained that “It would have just been really nice to see somebody that reminds me of me […] somebody that I can think ‘that'll be me in 10 years’ time’.” Another noted that, “If you can see someone else who is doing it, it makes you think you can do it more. But it’s all middle-aged white men […] I don’t relate to that at all.” Several suggested that role models were particularly valuable when they appeared to share similar backgrounds and experiences, with one participant explaining that *“*It's easier when it's somebody that is like me and has [overcome] the same barriers, or had to jump through the same hoops, and had to prove themselves that same extra little bit that you always seem to have to as a woman.”

The absence of such role models sometimes contributed to uncertainty about academic career progression. As one participant reflected, “Because there’s not that role model there […] there was always part of my brain being like ‘Why aren’t women making it that far? What's wrong with the career path?’”

Exposure to female role models in the wider research community was similarly limited. Participants described attending “conferences [where] I've been the only woman of about 80 people”. This lack of visibility extended beyond academia into popular media representations of women doing mathematics, with one participant commenting that she “can’t think of a single woman [in the media], apart from the woman on *Countdown*” (a UK game show featuring an arithmetic challenge).

#### Experiences of Microaggressions and Sexism

Participants described experiences of sexism and gender-based microaggressions during their PhD studies, many of which cannot be described in full to protect the participants’ anonymity. Incidents experienced and witnessed included inappropriate comments about their own or a female colleague’s appearance, sexual objectification, women being spoken over or dismissed in group settings, and the minimisation of women's academic achievements. One participant commented, “Microaggressions are things that happen, I don't want to say daily… but are things that happen.”

One of the most recognised forms of microaggression by participants involved the devaluation of women's accomplishments and the suggestion that opportunities were awarded based on gender rather than merit. Participants often referred to the concept of, and the fear of being, the “token woman”. One recalled being told by a male peer that her conference talk had only been accepted because “the conference is making an extra effort to accept women”. Such experiences were described as fostering self-doubt, with one participant reflecting that she had “the idea that ‘you've only got an interview because you're a woman and they're trying to be diverse, rather than because you actually deserve it’”. Another described a similar incident as “planting a seed” that led her to question “Am I here because of my work or am I here because of my gender?”

Participants also described more subtle forms of gender-based microaggression in their day-to-day research environments. These included being spoken over, infantilised, or judged differently from male colleagues. One participant described a colleague’s behaviour as “almost infantilising”, while another noted that a male colleague “had a tendency to speak over me”.

Notably, participants described experiencing gender-based microaggressions and sexism from colleagues at a range of career stages, including fellow PhD students and post-doctoral research associates. Female academics were perceived as being “mocked more than the male academics” and some male peers were described as “much more rude to females”. One participant recalled being mocked for having difficulties with the high-performance computing (HPC) facilities: “I couldn't get something to work, and it turns out [the HPC] itself was broken. And then one of [my peers] started mocking me for not being able to run my codes even though I knew it wasn't [my fault].”

Gendered judgements around appearance were also reported. Participants perceived negative attitudes towards women who did not conform to expected norms within mathematical sciences environments. One participant observed that “if a woman wears makeup or if a woman doesn't just wear like jeans and t-shirt […] I've seen negativity towards that before”. Another described a “well-intentioned” comment from her supervisor about her changing her hairstyle, explaining that “It’s not nasty, but it's just weird.”

Participants also identified examples where they perceived women to have fewer opportunities than male colleagues, particularly in relation to research visibility and speaking opportunities: “Guys would have been picked over girls for giving talks and whilst I want it to be that guys are just louder and therefore observed more easily, I do think there was some sexism behind it.”

Experiences of responses to these incidents were mixed. Some participants felt that incidents were minimised by colleagues or dismissed as isolated events. One participant described a group discussion in which a male peer stated that “sexism doesn’t exist”, while another said that they felt a more senior staff member had “tried to pass [a sexist incident] over as an isolated event”, which led to a sense of “disappointment in the system”. Although most participants were aware of reporting mechanisms, many had chosen not to report incidents. Some participants felt unsupported, with one explaining that her supervisor “wasn't good with that”, while others described receiving “a positive reaction from the institution”.

Participants also reflected on their own roles as potential active bystanders. One participant questioned whether she “should be more outspoken when [sexism] happens”. Although many expressed a desire to challenge sexism and “make things better”, this was often accompanied by a sense of powerlessness, with one participant reflecting that “If I do [say something], it’s not going to change.”

#### A Complex Picture of Professional Self-Identity

All participants described being confident in their mathematical abilities during secondary school. Most reported being “top of the class” and choosing to study mathematics at university because they were “good at it”. Despite this, there was a tendency to include self-effacing prefaces such as “I don’t know how to say it without sounding cocky…” when discussing their abilities.

Participants' experiences during university and doctoral study were more varied. While some retained a strong sense of confidence in their mathematical abilities, others described experiencing imposter syndrome. One participant reflected, “Every lecture I felt I didn’t understand… but then I would do well in my exams. Even by fourth year, when I knew I was doing well, I still felt like I was crap at it the whole time.”

Several participants described a decline in confidence as they transitioned from being “top of the class through school” to the university environment where “everybody is really good at maths”. Doctoral study was perceived as particularly challenging because there was an expectation that “you have to understand everything that you’re doing”. One participant explained, “I feel like I’m good at maths but […] I still feel like I’m stupid at what I do day to day.”

Participants frequently reflected on differences in how confidence was expressed by men and women. Many felt that men outwardly “appeared more confident than women”, even if this confidence was at times misplaced: “I’ve had multiple conversations with my male peers where you discuss a problem, and even if they’re wrong, they’re fairly confident.” Others questioned if “men are *actually* more confident, or if women are not so used to speaking up”. Some participants said female PhD students were more likely to “put themselves down” in front of a group and “make self-[deprecating] jokes which male PhD students don’t do”. One participant described internalised misogynistic ideas of intellectual inferiority as a barrier to developing their mathematical confidence: “People did used to think that women were just genetically worse at maths and physics somehow… that’s still in my mind somewhere I guess.” Another commented that “[Men] have confidence in their abilities more than their abilities should allow them to, and to be honest, I think that's a great thing. I think a lot of it is ‘pretend you belong until you actually do’, and I think the guys have got that from the get-go but the girls for some reason don't.”

Participants repeatedly referred explicitly to imposter syndrome when discussing their self-confidence and identity as a mathematician: “Until I get past [my] imposter syndrome I don't feel as though I can truly embed myself in the department.” One participant linked their imposter syndrome to feelings of difficulty in assessing progress: “It's hard with a PhD because there's no way of measuring yourself against your peers. When it was exams, you could see you're one of the better ones, or you're one of the worst ones […] but with a PhD you just don't have a clue. And everything is so hard […] so I just always [assume] that I am slow and I am not very good at it.”

Some participants perceived imposter syndrome to be “a much bigger issue amongst women”. One participant explained that “It's really common to have imposter syndrome, but I do think that being a woman makes it harder, because there's the societal things of the past of ‘women don't do science’ […] and ‘women have to stay at home’, so when you're the one going up against that, as well as the usual imposter syndrome, I think it's more.”

Questions of confidence and belonging extended into participants' professional identities as mathematicians and researchers. In exploring whether participants identified with the professional identities of “mathematician” and “researcher”, some cited imposter syndrome explicitly, while others described it implicitly in a feeling that they had not yet “done enough to warrant being a proper researcher”. One participant described feeling overtly excluded from the research community, which “feels like an elite club where the door is constantly being closed in my face”. Participants frequently felt that gender had negatively influenced the development of their professional identities.

Participants also described tensions between stereotypical images of mathematicians and femininity. Some felt that their appearance marked them as outsiders within mathematical sciences environments. One participant reflected that “constantly looking like you don't belong visually can impact your self-esteem”. Several participants reported modifying how they dressed and presented themselves in their working environment: “I quite like wearing makeup, but it seems like no one [else] wears makeup, so I don’t wear makeup for uni. If I decide to wear it, I feel paranoid. I feel like I’m being judged.”

#### The View of Gender as a Barrier to an Academic Career

Participants described a range of future career aspirations. Some hoped to (or had already) secured postdoctoral research positions, others planned to (or had already) moved into roles outside of academia, and some remained open to all pathways. For many, career plans had not been a major consideration when they first chose to study mathematics. Participants commented that “I was never set on anything career-wise” and “I haven’t always wanted to have a career in maths.”

Despite these differing aspirations, most participants felt that gender could act as a barrier to pursuing a long-term academic career. Several reflected that it would “be harder for [them] to succeed than a man” in academia and expressed concern about the compatibility of academic career with family life, particularly the challenges associated with temporary contracts, geographical mobility, and uncertainty surrounding maternity leave and caring responsibilities. One participant perceived a “lack of support” for women in academia and described family formation as “definitely a barrier”, while another felt that “the academic life isn’t suited to raising a child”. Similar concerns were expressed by a participant who reflected, “I am female and reaching the age where I do want kids and I don't think that's easy to do in academia.” Several participants also viewed the expectation of frequent relocation as incompatible with family life, commenting that pursuing an academic career might require them to “drag [a family] all over the world”, while one participant simply stated that she “just [doesn’t] want to move around every couple of years”.

Participants frequently linked these concerns to the lack of visible female representation within senior academic roles. For some, the underrepresentation of women contributed directly to doubts about their own prospects, with one participant commenting that “The male representation definitely made me think I probably won't make it.” Others described growing frustration with the “toxic” academic environment: “It's getting more and more frustrating to be a woman in maths, and I can see why a lot of my friends don't want to stay in academia.”

In contrast, industry was often perceived as offering a more supportive and stable alternative. Participants viewed STEM industries as being more proactive in addressing gender inequality and providing opportunities for women. One participant commented that “the private sector seems to be further ahead than universities in terms of giving opportunities to women”, while another felt that “in academia maybe women struggle a bit more now to survive, rather than in industry”. Another participant perceived that “[Industry] are very proactive about getting [EDI initiatives] done, and I feel like they actually care about equality.” Greater employment stability, improved work-life balance, and higher salaries were also mentioned as advantages of non-academic careers.

Not all participants felt that gender influenced their career decisions. Some viewed future choices primarily through the lens of opportunity and personal interest. As one participant explained, her decision “wouldn't be driven by the fact that I was a female […] it would be driven by the fact of what the opportunities in academia are”. Nevertheless, for many participants, concerns about gender and academic culture were closely tied to their future career plans. These push factors were powerfully summarised by one participant: “When I write pros and cons for leaving academia, most of the reasons to leave come from me being a woman.”

### Improving Experiences: Suggestions from Participants

Participants were invited to suggest actions that they feel would have improved their PhD experiences. While many welcomed existing EDI initiatives, others were sceptical of their impact, criticising them as “box-ticking” and “[failing to] reach a wider audience”. One participant explained that these activities and events were “mostly attended by the underrepresented group” and that she felt they “should be mandatory for the wider group to attend, so that they realise the issue”.

Participants also expressed frustration with the way some initiatives were delivered. Informal networking events were often viewed as insufficient, with some even seen to be reinforcing gender stereotypes. One participant described a women’s networking event as “tea and cake in a corridor”, commenting that it felt “awkward” and “like someone somewhere had gone, ‘What do women like? Women like cake!’”. Others highlighted practical barriers to organising meaningful activities, noting that events were frequently dependent on the efforts of individual staff members rather than being embedded within departmental structures: “It's always ran by an individual who cares rather than an actual school event.”

Despite these criticisms participants generally viewed EDI work as important and acknowledged that some staff were making genuine efforts to improve departmental culture. One participant described the department as “trying really hard”, while another felt that “there are people in the EDI committee that genuinely want to change things”. Despite this, there was still a perception that it is “hard to get things done”, with one participant describing her experience on the EDI committee as “arguing about admin for an hour rather than actually doing anything”.

Many participants felt that workshops focused on specific issues would be more beneficial than informal networking events. Several spoke positively of a previous workshop which focussed on combatting imposter syndrome, saying “more things like that would be super helpful”. Suggestions included facilitated focus groups, structured discussions around EDI topics, and talks from both internal and external speakers. Participants also noted that existing events were not always widely advertised.

Support structures were another area identified for improvement. While participants appreciated being signposted to central university services, some preferred support that was embedded within their department. One participant explained that “I don't want to ring a student helpline and talk to some stranger about it, I would much prefer to speak to someone in my department.”

Flexible working was highly valued, but experiences differed between supervisory teams. One participant explained, “for me personally I feel well supported and [appreciate] being able to work flexibly, but it probably depends on your supervisor”, noting that “some people’s supervisors want them to work in person all the time”. Another noted that “flexible working is really important” and that “[meetings and events] are usually within sensible working hours which allow for other commitments”. One participant suggested that academia could further normalise healthy work-life balance through measures such as encouraging staff and students to take all their allocated annual leave.

Finally, access to female role models and mentors was identified as a particularly important area for improvement. Participants valued the opportunity to speak with women who had navigated similar challenges, with one explaining that “Even if they weren’t a supervisor, any female member of staff that I could have gone to would have been nice.” More broadly, participants viewed improving gender representation within departments as a key mechanism for improving research culture. As one participant reflected, “If [the department] can address the gender split more in the staff, almost everything else will fix itself.”

## Discussion

This study explored the experiences of women undertaking doctoral study within a UK mathematical sciences department. Participants described challenges relating to belonging, support networks, professional identity development, and gendered experiences within academia. While many of these issues have been identified previously within broader STEM populations, the findings demonstrate their continued relevance within a UK mathematical sciences context and at a critical stage of the academic pipeline.

Although several distinct recurring themes were identified, participants' accounts suggest that these themes are closely interrelated rather than independent. For example, the underrepresentation of women within mathematical sciences can reduce exposure to female role models and mentors, which may in turn influence belonging, professional identity development, and perceptions of academia as a future career. Experiences of sexism and microaggressions further shaped participants' views of the research environment and, for some, contributed to concerns about progression within academia. Collectively, these findings suggest that decisions about future careers are shaped by an accumulation of experiences rather than any single factor.

### Improving Experiences

An important contribution of this study is the participant-led recommendations for improving research culture within the mathematical sciences. Participants highlighted the value of greater access to role models, mentoring opportunities, visible female academics, improved awareness of sexism and microaggressions, and more supportive approaches to work-life balance.

Increasing opportunities to engage with diverse role models may represent one of the most immediate and achievable interventions. Participants frequently emphasised the importance of seeing women successfully occupying academic positions and navigating careers within the mathematical sciences. Practical mechanisms for achieving this include seminar programmes, mentoring schemes, conference participation, and increased visibility of women across departmental activities. It is worth noting that there are limited data concerning the gender balance in specific mathematical disciplines, but in one US report there were four times more female doctoral recipients in statistics than applied mathematics [122]. Publication of data on the gender balance in undergraduate mathematics modules and fields of doctoral study in UK HEIs, and studies exploring the reasons for differences, could allow for more targeted gender-equality initiatives.

Participants also highlighted the importance of addressing sexism and gendered assumptions within academic environments. Their recommendations align with existing work advocating increased awareness of microaggressions, allyship, and evidence-based EDI initiatives. While many institutions have already implemented such programmes, our findings suggest that participants continue to perceive a need for greater discussion of these issues within doctoral training environments.

Finally, participants frequently discussed the importance of supportive attitudes towards personal relationships, caring responsibilities, and work-life balance. These findings reinforce ongoing efforts within higher education to develop family-friendly policies and flexible working practices. Participants viewed such initiatives not only as supporting wellbeing but also as influencing perceptions of whether an academic career is compatible with their future aspirations.

As in similar previous qualitative studies, many of our participants expressed that they were “pleased” that this study was taking place and reflected that discussing their experiences had been “helpful” and gave them a feeling of “being listened to”. This highlights that such qualitative research is valued by participants and motivates future work to create a continual dialogue with underrepresented groups in academia.

### Strengths and Limitations

This study involved a small sample of eight participants from a single UK HEI. As participation was voluntary, individuals with particularly positive or negative experiences may have been more likely to participate than those with more neutral views. Some participants expressed a personal interest in changing institutional norms, particularly around sexism and microaggressions. Consequently, the findings may not represent the full range of experiences of female doctoral students within the mathematical sciences. While caution should therefore be exercised when generalising the findings, many of the themes identified are consistent with previous research across STEM disciplines and within a range of institutional contexts, suggesting that the findings may be transferable to similar settings.

Exploring the intersection of gender with participants’ ethnicity, parental status, disability and other personal characteristics may have provided valuable insights. However, the small sample size limited the extent to which intersections with other social identities could be examined. Although this study was open to participants who identify as a gender minority (e.g., transgender, non-binary, gender fluid), all participants were cisgender female. Future research with larger and more diverse samples could examine these intersections in greater depth.

The analysis was undertaken by researchers with experience of studying and working within mathematical sciences environments. While this insider perspective provided valuable contextual understanding, it may also have influenced the interpretation of participants' accounts. In keeping with a reflexive thematic analysis approach, interpretations were developed through iterative discussion, repeated engagement with the data, and ongoing reflection on the researchers' own assumptions and experiences.

The use of semi-structured interviews means that responses may be shaped by interviewer prompts and the interactive nature of the interview process. This was mitigated with a common interview guide (Online Resource 2), open-ended questioning, neutral prompts, and reflexive awareness. We found that participants tended to answer the pre-interview Likert scale questions in a more positive or neutral manner than when discussing the same questions during the interview process. It is possible that participants came to recognise issues of sexism in these questions once they reflected on their own experiences in a psychologically safe interview process.

Finally, the participants in this study were all PhD students during the Covid-19 pandemic. Although previous research has suggested that the pandemic disproportionately affected women in academia [123], participants rarely identified Covid-19 as a major influence on their experiences beyond references to remote working. Nevertheless, it is possible that the broader circumstances of the period influenced participants' experiences in ways that were not explicitly recognised or discussed.

### Directions for Future Work

Future research should explore whether the themes identified in this study are observed more broadly across the mathematical sciences community in UK higher education. While the present study provides an in-depth account of doctoral students' experiences within a single department, a larger multi-institutional study would allow exploration of how experiences vary across different mathematical sciences departments and institutional contexts.

A larger sample would also make it possible to examine factors that could not be meaningfully explored within the scope of the present study, including potential differences associated with doctoral stage, nationality, ethnicity, disability, parental status, and other aspects of identity. Such work would provide a richer understanding of how gender intersects with other characteristics to shape experiences within mathematical sciences research environments.

The findings also highlight the value of further research focused specifically on the doctoral stage of the academic pipeline. Future qualitative studies could build on the themes identified here to explore particular issues in greater depth, including belonging, identity development, mentoring relationships, and career aspirations. Longitudinal research following doctoral students over time could also provide insight into how these experiences influence progression into postdoctoral, academic, and non-academic careers.

Finally, this work highlights the need for a national benchmarking study of doctoral students within mathematical sciences. Such work could establish the prevalence of the themes identified here, identify variation across institutions and disciplines, and provide an evidence base to inform future initiatives aimed at improving participation, wellbeing, and retention within the mathematical sciences.

## Conclusion

This study explored the lived experiences of female doctoral students within a UK mathematical sciences department. Participants' accounts highlighted the continuing importance of belonging, support networks, professional identity development, and gendered experiences in shaping doctoral study and future career aspirations. While many of these issues have been identified elsewhere in STEM, the findings demonstrate their relevance within the specific context of doctoral study in the mathematical sciences in a UK HEI.

By providing a mathematics-specific account of women's doctoral experiences and presenting participant-led recommendations for change, this study contributes to ongoing efforts to improve research culture and support women's progression within the mathematical sciences.

## Supplementary Information

Below is the link to the electronic supplementary material.

Supplementary file1 (DOCX 40 KB)

Supplementary file2 (DOCX 40 KB)
