# Supplementary material for: Voices of Women in Mathematical Sciences Higher Education: the PhD Student Experience
Source: Mathematica (N Y). 2026 Nov 4;5(4):75. doi: 10.1007/s44007-026-00256-3 (PMC13736337; doi:10.1007/s44007-026-00256-3)
Supplement: Supplementary file 1 [file 44007_2026_256_MOESM1_ESM.docx]

**Supplementary Information for *Voices of women in mathematical sciences Higher Education: the PhD student experience***

**Online Resource 1: Pre-interview questionnaire**

**Participant personal details:**

Name: Click or tap here to enter text.

Date of birth: Click or tap to enter a date.

Gender: Click or tap here to enter text.

Is this the gender you were assigned at birth: Click or tap here to enter text.

**Pre-PhD education details:**

Secondary/high school: Click or tap here to enter text.

A level (or equivalent qualifications) and grades: Click or tap here to enter text.

Undergraduate degree subject: Click or tap here to enter text.

Undergraduate degree institution: Click or tap here to enter text.

Undergraduate degree classification: Click or tap here to enter text.

**PhD education details:**

Date started PhD: Click or tap here to enter text.

Date PhD awarded (or estimate if current student): Click or tap here to enter text.

Funding length: Click or tap here to enter text.

Broad mathematics research area (e.g., pure, statistics, applied): Click or tap here to enter text.

Research group (e.g., quantum, astrophysics): Click or tap here to enter text.

**Post-PhD details/plans:** *If you have started work post-PhD please give details of your job description and the sector. If you have not yet graduated, please give details of your plans for future applications.*

Click or tap here to enter text.

During your interview, we’ll be discussing how you feel your gender may or may not have had an impact on your experience as a mathematics PhD student. The questions below give you some guidance on the themes we will be exploring. It may be helpful to spend some time thinking about these themes in advance of your interview, as well as answering any of the following questions which you feel comfortable doing.

**To what extent do you agree with the following statements? Please tick the appropriate box for each**

I feel that my gender has positively impacted my experiences as a mathematics PhD student.

| Strongly disagree | Disagree | Neutral | Agree | Strongly agree |
| --- | --- | --- | --- | --- |
|  |  |  |  |  |

I feel that my gender has negatively impacted my experiences as a mathematics PhD student.

| Strongly disagree | Disagree | Neutral | Agree | Strongly agree |
| --- | --- | --- | --- | --- |
|  |  |  |  |  |

I feel that my gender has played a positive role in my relationship with my PhD supervisors.

| Strongly disagree | Disagree | Neutral | Agree | Strongly agree |
| --- | --- | --- | --- | --- |
|  |  |  |  |  |

I feel that my gender has been a barrier to developing a good relationship with my PhD supervisors.

| Strongly disagree | Disagree | Neutral | Agree | Strongly agree |
| --- | --- | --- | --- | --- |
|  |  |  |  |  |

I feel that my gender has played a positive role in my relationship with my PhD peers.

| Strongly disagree | Disagree | Neutral | Agree | Strongly agree |
| --- | --- | --- | --- | --- |
|  |  |  |  |  |

I feel that my gender has been a barrier to developing a good relationship with my PhD peers.

| Strongly disagree | Disagree | Neutral | Agree | Strongly agree |
| --- | --- | --- | --- | --- |
|  |  |  |  |  |

I mostly feel confident in my mathematics abilities within my field.

| Strongly disagree | Disagree | Neutral | Agree | Strongly agree |
| --- | --- | --- | --- | --- |
|  |  |  |  |  |

I feel that I am a mathematician.

| Strongly disagree | Disagree | Neutral | Agree | Strongly agree |
| --- | --- | --- | --- | --- |
|  |  |  |  |  |

I feel that I am a researcher.

| Strongly disagree | Disagree | Neutral | Agree | Strongly agree |
| --- | --- | --- | --- | --- |
|  |  |  |  |  |

I feel that my gender has played a positive role in my mathematician/researcher identity.

| Strongly disagree | Disagree | Neutral | Agree | Strongly agree |
| --- | --- | --- | --- | --- |
|  |  |  |  |  |

I feel that my gender has been a barrier to feeling like a mathematician/researcher.

| Strongly disagree | Disagree | Neutral | Agree | Strongly agree |
| --- | --- | --- | --- | --- |
|  |  |  |  |  |

I feel an appropriate sense of workplace ‘belonging’ within the department.

| Strongly disagree | Disagree | Neutral | Agree | Strongly agree |
| --- | --- | --- | --- | --- |
|  |  |  |  |  |

I feel that my gender has played a positive role in feeling a sense of ‘belonging’ within the department.

| Strongly disagree | Disagree | Neutral | Agree | Strongly agree |
| --- | --- | --- | --- | --- |
|  |  |  |  |  |

I feel that my gender has been a barrier to feeling a sense of ‘belonging’ within the department.

| Strongly disagree | Disagree | Neutral | Agree | Strongly agree |
| --- | --- | --- | --- | --- |
|  |  |  |  |  |

My PhD studies have been affected by my family/caring commitments.

| Strongly disagree | Disagree | Neutral | Agree | Strongly agree |
| --- | --- | --- | --- | --- |
|  |  |  |  |  |

I feel that I have experienced micro-aggressions related to my gender during my PhD studies.

| Strongly disagree | Disagree | Neutral | Agree | Strongly agree |
| --- | --- | --- | --- | --- |
|  |  |  |  |  |

I feel that I have experienced sexism during my PhD studies.

| Strongly disagree | Disagree | Neutral | Agree | Strongly agree |
| --- | --- | --- | --- | --- |
|  |  |  |  |  |

I feel that it is important to me to see role models of my gender within mathematics.

| Strongly disagree | Disagree | Neutral | Agree | Strongly agree |
| --- | --- | --- | --- | --- |
|  |  |  |  |  |

I feel that I have been exposed to a good number of positive role-models of my gender within the department.

| Strongly disagree | Disagree | Neutral | Agree | Strongly agree |
| --- | --- | --- | --- | --- |
|  |  |  |  |  |

I feel that my gender has played a role in my post-PhD plans.

| Strongly disagree | Disagree | Neutral | Agree | Strongly agree |
| --- | --- | --- | --- | --- |
|  |  |  |  |  |

I feel that my gender would make it more difficult for me to pursue an academic career.

| Strongly disagree | Disagree | Neutral | Agree | Strongly agree |
| --- | --- | --- | --- | --- |
|  |  |  |  |  |

I feel that my gender would make it more difficult for me to pursue a career outside of academia.

| Strongly disagree | Disagree | Neutral | Agree | Strongly agree |
| --- | --- | --- | --- | --- |
|  |  |  |  |  |

Prior to this study, I have thought about how my gender has impacted my professional experiences.

| Strongly disagree | Disagree | Neutral | Agree | Strongly agree |
| --- | --- | --- | --- | --- |
|  |  |  |  |  |

In your interview we will be discussing the themes touched on above, but we are interested in hearing about anything else you feel has had an impact on your thoughts and experiences that we may not have considered. If there are any other topics you feel are related to your gender and experiences/choices as a mathematics PhD student that you would be willing to discuss, please note them here:

Click or tap here to enter text.

Is there any additional support you feel you could have been offered that would have improved your experiences as a woman or gender-minority PhD student in the department?

Click or tap here to enter text.
