# Supplementary material for: Voices of Women in Mathematical Sciences Higher Education: the PhD Student Experience
Source: Mathematica (N Y). 2026 Nov 4;5(4):75. doi: 10.1007/s44007-026-00256-3 (PMC13736337; doi:10.1007/s44007-026-00256-3)
Supplement: Supplementary file 2 [file 44007_2026_256_MOESM2_ESM.docx]

**Supplementary Information for *Voices of women in mathematical sciences Higher Education: the PhD student experience***

**Online Resource 2: Interviewer crib sheet**

Below we provide in full the sheet developed to support the researchers in conducting semi-structured interviews with participants. This was developed with the support of a university equality, diversity and inclusion representative. Not all the questions below were asked of every participant. The interview guide was intended to support discussion rather than prescribe it, and participants were encouraged to introduce topics they felt were relevant to their experiences.

**Interview Guidance**

The aim of this interview is to explore the participants thoughts, feelings, and personal experiences of how they feel their gender has impacted their experience as a mathematical sciences PhD student. Some interviewees may have lots to say already, whereas some may need more guidance. Hopefully the pre-interview questionnaire will have provided all with some time to think about the issues that we will raise.

This document is to serve as a guide for structuring the interviews covering the key themes identified as relevant from previous literature in a sensible order. Note the participants may have their own ideas, and it’s our job to try and extract them! This is all about listening to the participants experiences and ideas, there are no ‘right or wrong’ answers, and so we must take care to avoid leading questions and those which could be seen as having an underlying value judgement. Please also be aware of the sensitive nature of some of the themes raised and respond in an objective, professional and compassionate manner to any particularly personal issues that are discussed.

**Safeguarding:** We should be prepared for participants telling us about experiences that could relate to abuse, including gender-based violence, discrimination, harassment, bullying and hate crime. In this case we should listen to the participant, respond sensitively, and signpost the relevant services (most likely, University Student/Staff Wellbeing or their GP services). If we feel the participant’s safeguarding is at risk, we may need to breach the confidentiality agreement to escalate the issue – this will be mentioned to the participants in the introduction.

**Gender Inclusivity:** For brevity in this document and the project title we sometimes use ‘women’ only, however the full group of interest is ‘women and otherwise gender minorities within mathematics’ (this wording is used on all the participant forms) and would include transgender, non-binary, gender fluid and any other self-identifying participants other than cis-male (the majority gender). We will know how the participant identifies from their pre-interview questionnaire – so keep this in mind throughout and use the relevant pronouns (please check with the participant if you are unsure).

**Feedback to the participant:** There are times when we may want to give feedback/responses to the participants – it is important that when this is done is it based on neutral observations rather than our own beliefs and preferences. Feedback should be used to: bring about understanding, praise (e.g., if the participant is unable to see their positive contributions/progress of best practice), encourage, inform, raise awareness. Feedback is not: giving advice, giving solutions, directing/telling, giving opinions, or making judgements.

**Setting the tone:** We aim to use a relaxed and informal tone to promote inclusion and open discussion. We should give the participants plenty of opportunities to guide the discussion and bring up any additional points or questions they have. We should follow up on points they raise (e.g., “and how did that make you feel?” “What was that like?”) and keep encouraging them to share any other experiences or ideas they’d like to share (“Is there anything else you’d like to share at this point?” “Is there anything else you’d like to share that we haven’t mentioned yet?”)

**Start of the interview (0-5mins)**

- Introduce yourself to the participant and thank them for taking part in this study.
- Summarise the aim of the study again and signpost the structure of the interview (i.e., the interview should take approximately 1 hour, and I will be asking you about your personal experiences with gender in relation to your mathematical studies and career).
- Ask if they have any questions.
- Inform them they are free to **stop or pause** the interview at any time.
- Reiterate that anything discussed during the interview is part of a scientific study and will remain confidential, used for research purposes only.
- Inform the participant that this confidentiality will need to be breached if we feel there is an immediate safeguarding risk that we need to escalate.
- **Confirm they are happy to begin and start the meeting recording.**

**Part 1: Personal background**

*This section aims to get the participant comfortable talking and find out some more about their personal background.*

Sample Questions:

1. Can you tell me a bit about yourself? How long have you lived in this part of the UK?
2. Can you tell me a bit about your PhD project and how far through your studies you are? How are you finding it?

**Part 2: Pre-university experiences**

*This section aims to explore their experience leading up to their choice to study mathematics at university. We know already that the high-school experience has a big impact on higher education choices – here we want to explore how their gender impacted their view of mathematics while at school and their choice to study maths at the HE level.*

Sample Questions:

1. What was your experience like in maths at primary and secondary school?
2. Did you enjoy maths at school?
3. Did you feel you did well at maths at school?
4. What about physics and other science subjects?
5. Did you ever feel your gender played a role in those experiences in any way? What was the gender split like in your maths classes at school? How did this make you feel?
6. Are there any experiences that stand out as pivotal in your mathematics schooling? *(This could be anything to do with exams, teachers, other students etc).*
7. How did you decide to apply for mathematics at university?
8. Were you aware of what the gender split might be like in mathematics at university? Was this something that was important to you?
9. Did you feel there were any barriers or things you thought were off-putting about applying for mathematics at university? Was there anything that helped you overcome those barriers?
10. Did you have any worries about what doing a maths degree would be like?
11. Did you think about what you would do after your maths degree?

**Part 3: Under-graduate experiences**

*This section aims to explore their experience as a mathematics UG student (some of these will have been at this university, some will have been elsewhere, please see the participant’s pre-interview questionnaire).*

Sample Questions:

1. Where did you complete your UG studies?
2. What course did you do?
3. MMath experience: Did you always want to complete your Masters?/Were you always signed up to the 4 year MMath course? If yes, why? If no, what made you change your mind and when did this change happen?
4. Did you enjoy your undergraduate experience?
5. What were the gender splits like in your undergraduate classes? How did this make you feel? Were the gender splits different across modules? How did this affect the dynamic of the cohort?

**Part 4: PhD experiences**

*This section aims to explore their experiences as a PhD student in the department (note that some of the participants will have moved across the country or even the world to come and study their PhD at this university, whilst some may have completed their UG studies here). You may want to signpost to participants that we’re going to now be discussing their PhD experiences in this department and remind them that whatever they say is confidential to encourage them to be as open as possible.*

Sample Questions:

1. What made you decide to do a maths PhD? What about at this university? (i.e., did you apply for anywhere else? What attracted you to this university in particular?).
2. Have you enjoyed your PhD studies so far? / Did you enjoy your PhD studies?
3. Did your gender play any kind of role in your thoughts and feelings about making the decision to do a maths PhD?
4. What is/was the gender split like amongst PhD students? How does this make you feel? Is this a change from at UG?

**Part 5: Relationships with teachers/tutors/supervisors**

*This section aims to explore their relationship with their teachers, tutors, and PhD supervisors throughout their mathematics career. (You may have naturally touched upon some of these questions already in parts 1-3). A lot of the participants will have all male supervisory teams.*

Sample Questions:

1. What were your maths teachers like at secondary school? (Did you have many female/gender-minority maths teachers?)
2. What were your undergraduate lecturers like? How many of them were female/gender minority?
3. Did you feel well supported by your personal tutor and lecturers during your UG studies?
4. How is your relationship with your PhD supervisor/s? Do you have another mentor aside from your supervisors? (And how is that relationship?)
5. Do you feel your gender has affected your relationship with your PhD supervisors in any way? (If they are all male, how does this make you feel? Do you think you would (have) benefit(ed) from having a female supervisor? If they are female/gender minority, how does this make you feel? Do you think you have benefited from having a female supervisor?)
6. Do you feel well supported by your supervisor in terms of your career development?
7. Do you feel you trust and respect your supervisor? And do you feel they trust and respect you?

**Part 6: Role models and mentors**

*This section aims to the participants relationship with role models and their mentors (these may be personal or professional).*

Sample Questions:

1. Do you feel you’ve had some good role models throughout your mathematics career? (This could be at any time and may not be in a professional capacity). Were any of these role models female (or another gender-minority in mathematics)?
2. Do you think having female role models within mathematics is important to you? If yes, why do you think that is the case (i.e., do you have any ideas about how this would help you/why you would benefit/why it is important?) If no, why do you think that is the case (i.e., do you think role models are important in general? What makes you feel that you don’t require role models of your gender?)
3. Do you have/did you have a mentor outside of your PhD supervisors for approaching with personal issues or career advice? If yes, was this person of the same gender as you? Do you think this is important/how did this make you feel? If no, would you have liked to have a mentor outside of your PhD supervisors? Do you think their gender would play a role in your relationship?
4. Does/did the department provide opportunities for you to engage with diverse role models/speakers?
5. Has anyone else particularly impacted your maths career?
6. Which women or gender-minorities in mathematics inspire you? (or have inspired you?)
7. Do you think mentoring has had a positive impact on your career and/or personal development?

**Part 7: Sense of belonging**

*This section aims to explore their feeling of “belonging” to both the department PhD cohort and the wider mathematical community. We want students to feel they belong as their authentic selves, not to just “fit in” so there are some subtleties here.*

Sample Questions:

1. Do you feel a sense of belonging within the mathematics PhD cohort? (Would you say you “fit in”? Have you made any conscious changes to feel you “fit in” within the cohort?)
2. Do you feel a sense of belonging within the wider department? (Would you say you “fit in”? Have you made any conscious changes to feel you “fit in” within the department?)
3. Does your gender make you feel like you belong any more or less to the PhD cohort or the wider department?
4. Would you describe yourself as a mathematician? (When do you think you started to?/When do you think you will?)
5. Do you think your gender has impacted your sense of feeling like a mathematician?
6. Would you describe yourself as a researcher? Do you think your gender has impacted your researcher identity?
7. Do you feel your contributions are valued within your team/your research group/the department?
8. What do you think it means to be a woman in mathematics?

**Part 8: Confidence**

*Confidence is a theme that comes up repeatedly in the literature, with evidence to suggest women are less confident in their mathematical abilities than men (even at the PhD level). Note that this is not a suggestion that women have a lower attainment - we are working on the assumption that this difference in confidence is unfounded. In this section we want to explore how our participants feel about their mathematical abilities (keeping in mind that they are studying for a PhD so definitely have great ability).*

Sample Questions:

1. How would you describe your mathematics ability during your schooling years? What about at undergraduate? Have you always felt “good at maths”? Has your confidence in maths changed over time?
2. How would you describe your mathematics ability now?
3. Do you think men are more confidence in their mathematical abilities than women? If so, what has made you think that is the case?
4. Did you feel confident to put your hand up and answer/ask questions in lectures? Do you feel confident now in approaching your supervisors/mentors if you have questions?
5. Do you feel confident in expressing your opinions and ideas with your supervisors and peers?

**Part 9: Micro-aggressions and sexism.**

*This section aims to explore any experiences with anything from micro-aggressions, sexism, and harassment. Note that this section may be uncomfortable for participants if they do have experiences to share (see safeguarding notes).*

Sample Questions:

1. Do you have any personal experiences with micro-aggressions related to your gender within the department? What about in your education before your PhD? How did these experiences make you feel?
2. Do you feel you’ve ever witnessed micro-aggressions towards other PhD students or members of staff related to gender within the department? Can you tell us about that? How did witnessing this make you feel?
3. Do you have any experiences with over sexism, discrimination or harassment related to your gender within the department? What about in your education before your PhD? How did these experiences make you feel?
4. Have you ever witnessed overt sexism, discrimination or harassment towards another PhD student or member of staff related to their gender within the department? Can you tell us about that? How did witnessing this make you feel?
5. If yes to any of the above: Do you know if the event was reported? Do you feel the institution responded appropriately? Do you think more could be done to stop events like these taking place within the department?
6. Do you know where to report bullying, harassment, hate crime etc?

**Part 10: Family, other commitments, and mental health**

*This section aims to explore if other aspects of the participant’s life have had an impact on their PhD studies – such as family commitments, caring responsibilities, mental health problems. Some research suggests women disproportionately suffer in their careers due to taking on a greater responsibility of family and caring roles within their personal lives, as well as barriers relating to maternity leave and career breaks.*

Sample Questions:

1. On your questionnaire you note that you have had some family or caring commitments that have affected your PhD studies, can you tell us a bit more about this?
2. Do you think women are disproportionately affected by family and caring commitments such as these?
3. Do you feel the department has been supportive of you throughout your experiences with your family/caring commitments?
4. Do you feel the timings of meetings and events takes into consideration work life balance?
5. Do you feel your mental health and wellbeing are supported?
6. Do you feel the department supports flexible working? Is flexible working something that is important to you?

**Part 11: Future career**

*This section aims to explore their decisions post-PhD. Some participants will have already started their post-PhD roles, some will be in the process of applying for opportunities, and some may not have started or thought about it at all.*

Sample Questions:

1. If working already: What is your current role? How did you decide to go into this area? When did you decide that this would be your next step after your PhD?
2. If still PhD student: Do you have any ideas about what you would like to apply for after your PhD? (Could be types of roles, interested in a particular sector or industry etc). Why does this appeal to you? When did you decide this would be your ideal next step?
3. Do you think your gender has had any impact on your decision to stay in/leave academia?
4. Do you think your gender would make it more difficult for you to pursue an academic career? Do you think your gender would make it more difficult for you to pursue an industry career? Why do you think that is the case?
5. Do you see any barriers to your continuing on your chosen career pathway? (Do you feel any of these relate to your gender?)

**End of the interview**

- Ask the participant if there is anything else they would like to add/talk about.
- Signpost that you’ll now draw the interview to a close and thank the participant for their time.
